# Fourth mRNA COVID-19 vaccination in immunocompromised patients with haematological malignancies (COBRA KAI): a cohort study

**DOI:** 10.1016/j.eclinm.2023.102040

**Published:** 2023-06-15

**Authors:** Quincy Hofsink, Sabine Haggenburg, Birgit I. Lissenberg-Witte, Annoek E.C. Broers, Jaap A. van Doesum, Rob S. van Binnendijk, Gerco den Hartog, Michel S. Bhoekhan, Nienke J.E. Haverkate, Johan van Meerloo, Judith A. Burger, Joey H. Bouhuijs, Gaby P. Smits, Dorine Wouters, Ester M.M. van Leeuwen, Hetty J. Bontkes, Neeltje A. Kootstra, Sandra Vogels-Nooijen, Nynke Rots, Josine van Beek, Mirjam H.M. Heemskerk, Kazimierz Groen, Tom van Meerten, Pim G.N.J. Mutsaers, Marit J. van Gils, Abraham Goorhuis, Caroline E. Rutten, Mette D. Hazenberg, Inger S. Nijhof, COBRA KAI study team, Iris M.J. Kant, Thecla Graas, Belle Toussaint, Sterre de Jong, Shahan Darwesh, Sandjiv S. Mahes, Dora Kamminga, Matthijs Koelewijn, Gino Faber, Guus Beaumont, Marije D. Engel, R. Cheyenne N. Pierie, Suzanne R. Janssen, Gino Faber, Edith van Dijkman, Jarom Heijmans, Yara Y. Witte, Rogers A. Nahui Palomino, Said Z. Omar, Sonja Zweegman, Arnon P. Kater, Caya van den Vegt, Ilonka Arends-Halbesma, Emma de Pater, Margriet J. Dijkstra, Nynke Y. Rots, Esther Siteur-van Rijnstra, Dennis M. de Rooij, Rogier W. Sanders, Meliawati Poniman, Wouter Olijhoek, Jacqueline van Rijswijk, Tim Beaumont, Lusia Çetinel, Louis Schellekens, Yvonne M. den Hartogh, Jacqueline Cloos, Suzanne S. Weijers, Saïda Tonouh-Aajoud, Selime Avci, Elianne Roelandse-Koop, Willem A. Dik

**Affiliations:** a Department of Haematology, Amsterdam UMC Location University of Amsterdam, Amsterdam, Netherlands; b Amsterdam Institute for Infection and Immunity, Amsterdam UMC, Amsterdam, Netherlands; c Department of Epidemiology and Data Science, Amsterdam UMC Location Vrije Universiteit, Amsterdam, Netherlands; d Department of Haematology, Erasmus MC Cancer Institute, Rotterdam, Netherlands; e Department of Haematology, University Medical Centre Groningen, University of Groningen, Groningen, Netherlands; f Centre for Immunology of Infectious Diseases and Vaccines, National Institute for Public Health and the Environment, Bilthoven, Netherlands; g Laboratory of Medical Immunology, Radboud University Medical Centre, Nijmegen, Netherlands; h Department of Experimental Immunology, Amsterdam UMC Location University of Amsterdam, Amsterdam, Netherlands; i Department of Haematology, Amsterdam UMC Location Vrije Universiteit, Amsterdam, Netherlands; j Cancer Centre Amsterdam, Amsterdam UMC, Amsterdam, Netherlands; k Department of Medical Microbiology and Infection Prevention, Amsterdam UMC, University of Amsterdam, Amsterdam, Netherlands; l Central Diagnostic Laboratory, Amsterdam UMC, Amsterdam, Netherlands; m Department of Clinical Chemistry, Laboratory Medical Immunology, Amsterdam UMC, Amsterdam, Netherlands; n Netherlands Comprehensive Cancer Organisation, Utrecht, Netherlands; o Department of Haematology, Leiden UMC, Leiden, Netherlands; p Department of Infectious Diseases, Amsterdam UMC Location University of Amsterdam, Amsterdam, Netherlands; q Department of Haematopoiesis, Sanquin Research, Amsterdam, Netherlands; r Department of Internal Medicine-Haematology, St. Antonius Hospital, Nieuwegein, Netherlands

**Keywords:** Antibody response, SARS-CoV-2, COVID-19 vaccination, Booster vaccination, Haematological malignancies, Immunocompromised

## Abstract

**Background:**

Patients with haematological malignancies have impaired antibody responses to SARS-CoV-2 vaccination. We aimed to investigate whether a fourth mRNA COVID-19 vaccination improved antibody quantity and quality.

**Methods:**

In this cohort study, conducted at 5 sites in the Netherlands, we compared antibody concentrations 28 days after 4 mRNA vaccinations (3-dose primary series plus 1 booster vaccination) in SARS-CoV-2 naive, immunocompromised patients with haematological malignancies to those obtained by age-matched, healthy individuals who had received the standard primary 2-dose mRNA vaccination schedule followed by a first booster mRNA vaccination. Prior to and 4 weeks after each vaccination, peripheral blood samples and data on demographic parameters and medical history were collected. Concentrations of antibodies that bind spike 1 (S1) and nucleocapsid (N) protein of SARS-CoV-2 were quantified in binding antibody units (BAU) per mL according to the WHO International Standard for COVID-19 serological tests. Seroconversion was defined as an S1 IgG concentration >10 BAU/mL and a previous SARS-CoV-2 infection as N IgG >14.3 BAU/mL. Antibody neutralising activity was tested using lentiviral-based pseudoviruses expressing spike protein of SARS-CoV-2 wild-type (D614G), Omicron BA.1, and Omicron BA.4/5 variants. This study is registered with EudraCT, number 2021-001072-41.

**Findings:**

Between March 24, 2021 and May 4, 2021, 723 patients with haematological diseases were enrolled, of which 414 fulfilled the inclusion criteria for the current analysis. Although S1 IgG concentrations in patients significantly improved after the fourth dose, they remained significantly lower compared to those obtained by 58 age-matched healthy individuals after their first booster (third) vaccination. The rise in neutralising antibody concentration was most prominent in patients with a recovering B cell compartment, although potent responses were also observed in patients with persistent immunodeficiencies. 19% of patients never seroconverted, despite 4 vaccinations. Patients who received their first 2 vaccinations when they were B cell depleted and the third and fourth vaccination during B cell recovery demonstrated similar antibody induction dynamics as patients with normal B cell numbers during the first 2 vaccinations. However, the neutralising capacity of these antibodies was significantly better than that of patients with normal B cell numbers after two vaccinations.

**Interpretation:**

A fourth mRNA COVID-19 vaccination improved S1 IgG concentrations in the majority of patients with a haematological malignancy. Vaccination during B cell depletion may pave the way for better quality of antibody responses after B cell reconstitution.

**Funding:**

The 10.13039/501100001826Netherlands Organisation for Health Research and Development and 10.13039/100019573Amsterdam UMC.

**Research in context**

**Evidence before this study**

We searched PubMed on April 10, 2023, for English-language studies evaluating humoral immunogenicity of COVID-19 vaccines in patients with haematological malignancies, using the search terms “immunogenicity” or “antibody response”, “COVID-19 vaccine” or “SARS-CoV-2 vaccine”, and “haematology” or “cancer”. Reflecting the diverse palette of immune disorders observed among patients with haematological malignancies, vaccine immunogenicity was very heterogeneous in this population. On average, the primary 2-dose mRNA vaccination schedule induced lower SARS-CoV-2 spike protein (S) specific antibody concentrations compared to healthy individuals. We and others demonstrated that supplementation of the primary 2-dose schedule with a third vaccination significantly enhanced S1 IgG concentrations. Moreover, the third vaccination led to antibody maturation, as reflected by an improved virus neutralising capacity per antibody. Nevertheless, a substantial part of patients with haematological malignancies remained to have significantly lower S1 IgG concentrations compared to 2-dose-vaccinated healthy individuals.

**Added value of this study**

In this unique cohort of >700 immunocompromised patients with haematological malignancies, we demonstrated that a fourth mRNA COVID-19 vaccination significantly improved humoral immunity against SARS-CoV-2. Vaccination in absence of B cells primed the immune system and led to enhanced antibody maturity in response to booster vaccinations given after recovery of the B cell pool.

**Implications of all the available evidence**

Our data argue for continued vaccination, even in patients with B-cell depletion and in patients that are persistently immunocompromised. Vaccine effectiveness studies that focus on patients with a specific type of malignancy and stage of therapy are required to determine the consequences of the observed suboptimal antibody responses on COVID-19-related morbidity and mortality.

## Introduction

Patients with haematological malignancies who are immunocompromised due to the malignancy itself or the treatment thereof remain at risk for COVID-19-related morbidity and mortality, despite the introduction of COVID-19 vaccines.^1^, ^2^, ^3^, ^4^, ^5^ Reflecting the diverse palette of immune disorders observed among these patients, vaccine immunogenicity was very heterogeneous in this population.^6^, ^7^, ^8^ On average, the primary 2-dose mRNA vaccination schedule induced lower SARS-CoV-2 spike protein (S) specific antibody concentrations compared to healthy individuals.^6^^,^^9^, ^10^, ^11^ We and others demonstrated that with a third vaccination, the majority of patients with a haematological malignancy did reach S1 IgG concentrations not significantly lower than those obtained by healthy, age-matched individuals after 2 vaccinations.^7^^,^^12^^,^^13^ Moreover, the third vaccination led to antibody maturation, as reflected by an improved virus neutralising capacity per antibody.^7^ This maturation was most pronounced for the Omicron variant and is similar as observed in healthy individuals.^14^ These findings supported the policy to standardise the primary COVID-19 vaccination series as a 3-dose schedule for patients with immunodeficiencies, including patients with haematological malignancies, which has now been implemented world-wide.^15^

Nevertheless, a substantial part of patients with haematological malignancies remained to have significantly lower S1 IgG concentrations compared to 2-dose-vaccinated healthy individuals.^7^ The observation that with each dose S1 IgG concentrations improved, despite ongoing immunodeficiencies, raised the question whether a fourth vaccination could further improve antibody concentrations and neutralising capacity of these antibodies.^16^^,^^17^

In this study, we aimed to compare antibody concentrations after 4 mRNA vaccinations (3-dose primary series plus 1 booster vaccination) in patients with a haematological malignancy with those obtained by age-matched, healthy individuals who had received the standard primary 2-dose mRNA vaccination schedule, also followed by a first booster mRNA vaccination.

## Methods

### Study design and participants

In this cohort study (COBRA KAI study) we analysed antibody responses to a 4-dose mRNA COVID-19 vaccination schedule in 16 pre-defined cohorts of patients with haematological malignancies, as described previously and in the study protocol available as a supplementary file with the online version of this article.^6^^,^^7^ In the current analysis, we evaluated all patients who received a fourth COVID-19 vaccination between December 2021 and April 2022, according to the Dutch National Institute for Public Health and the Environment guidelines, and were SARS-CoV-2 naive, as defined by N IgG <14.3 BAU/mL at all time points. Reference antibody concentrations were obtained from age-matched, healthy individuals who received 2 mRNA-1273 vaccinations followed by 1 booster vaccination with BNT162b2. Healthy individuals were all participants of the Vaccines and InfecTious diseases in the Ageing popuLation (VITAL) project, a public-private consortium to investigate infectious diseases and the effects of vaccination coordinated by the Dutch National Institute for Public Health and the Environment.^18^ Study protocols were approved by the Institutional Review Board of Amsterdam UMC location Vrije Universiteit and participating centres (COBRA KAI study; EudraCT 2021-001072-41) and Utrecht University (VITAL cohort; EudraCT 2019-000836-24). All patients provided written informed consent. Given the observational nature of this cohort study, randomisation and blinding was not applicable.

### Antibody concentrations and in vitro virus neutralisation

Prior to and 4 weeks after each vaccination, we collected peripheral blood, demographic parameters, and medical history including comorbidities and immunosuppressive therapy. Concentrations of antibodies that bind S1 and nucleocapsid protein (N) of SARS-CoV-2 were quantified in binding antibody units (BAU) per mL according to the WHO International Standard for COVID-19 serological tests, as described previously.^6^^,^^7^ Seroconversion was defined as an S1 IgG concentration >10 BAU/mL and a previous SARS-CoV-2 infection as N IgG >14.3 BAU/mL.^6^^,^^7^^,^^19^^,^^20^ Antibody neutralising activity was tested using lentiviral-based pseudoviruses expressing S of SARS-CoV-2 wild-type (D614G), Omicron BA.1, and Omicron BA.4/5 variants as described previously.^21^ As a measure for neutralising activity, the reciprocal dilution of sera required to inhibit viral infection by 50% (ID_50_) was determined by pseudovirus neutralisation assays in randomly selected study participants who obtained S1 IgG concentrations above 50 BAU/mL after the primary 2-dose vaccination schedule. Neutralising capacity per binding antibody unit was calculated by the ratio of ID_50_ to the binding S1 IgG concentration.

### Statistical analysis

Sample size calculation as applied for the study design has been described previously.^6^ In this previous analysis, we dichotomised the antibody response to below or above 300 BAU/ml on which the sample size calculation was based. Since then, it has become clear that the lower limit of S1 IgG concentrations that are considered sufficient to allow protection against severe COVID-19 vary depending on the specific variant of concern (VOC) and that the lower limit of 300 BAU/ml cannot be applied universally. For this reason, in this current analysis, we analysed antibody responses as continuous values. The sample size calculation is no longer applicable due to these VOC-dependent changes in interpretation of antibody response (i.e. continuously rather than dichotomously). Statistical significance for differences within groups (e.g. 3rd vs. 4th vaccination response) was calculated by a paired sample t-test for normally distributed data or a Wilcoxon Signed Rank test for non-normally distributed data. Statistical significance for differences between groups (e.g. patients vs. healthy individuals) was calculated by a Mann–Whitney U test for non-normally distributed data. Pearson's correlation was calculated between S1 IgG concentrations and ID_50_ after ^10^log transformation of both. Post-hoc analyses were performed to determine differences in antibody concentrations and neutralising capacity between never B cell depleted patients and B cell reconstituting patients. No sensitivity analyses were performed. Two-sided p-value <0.05 was considered statistically significant, where subcohort analyses were informal and exploratory. Statistical analyses were performed using the IBM SPSS Statistics for Windows, Version 28.0 (IBM Corp., Armonk, NY).

### Role of the funding source

The funders of the study had no role in study design, data collection, data analysis, data interpretation, or writing of the report.

## Results

### Antibody concentration and virus neutralisation

**Fig. 1:**
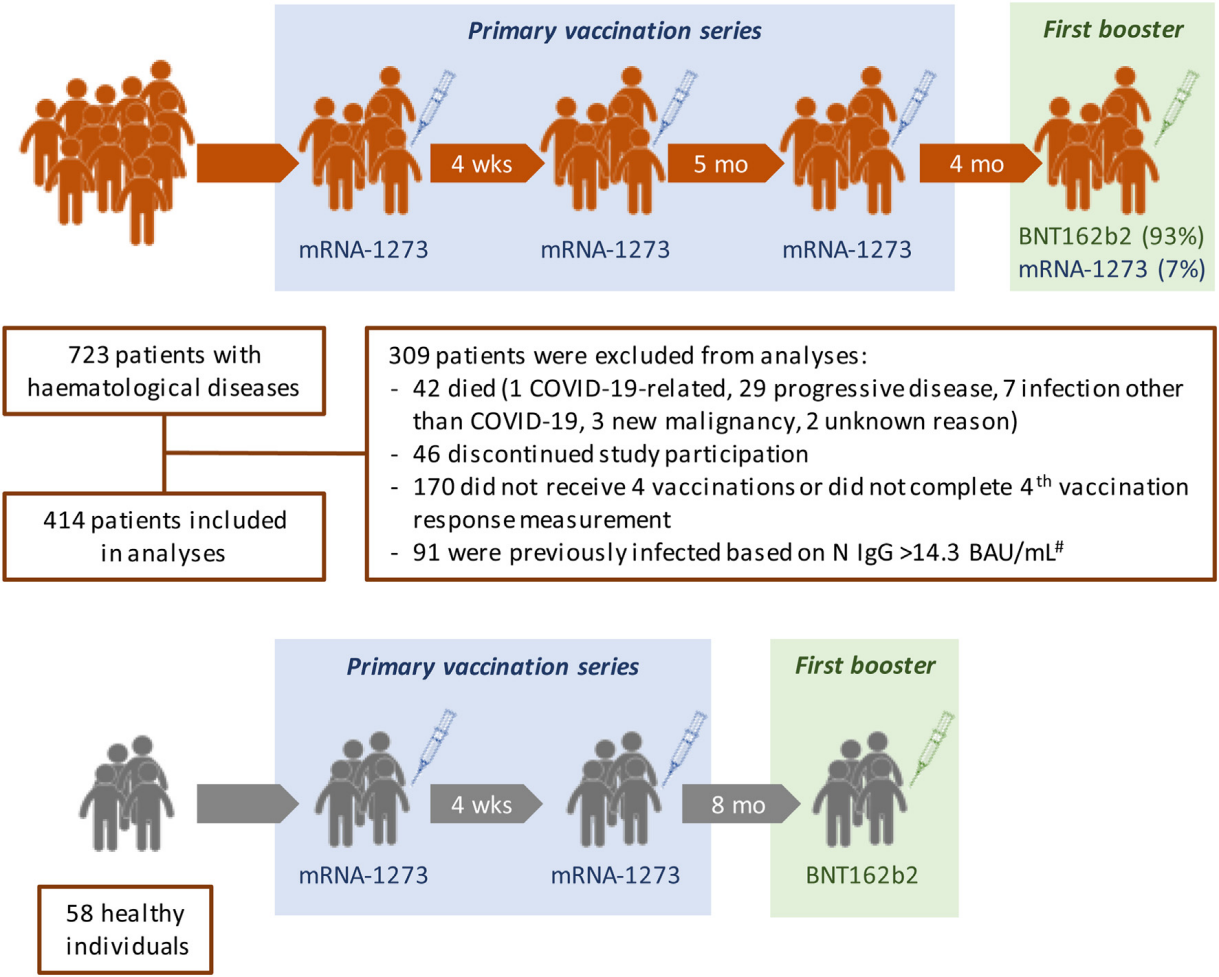
**Study participants and vaccination schedules.** 723 patients were included in this cohort study,^6^ of whom 414 met the criteria for fourth vaccination response analyses. Timing of vaccinations was according to the COVID-19 vaccination policy for immunocompromised patients governed by the Dutch Minister of Health (wks: weeks; mo: months). All participants received the primary 3-dose mRNA-1273 vaccination series, followed by a booster (fourth) mRNA vaccination (BNT162b2 in 93% of patients and mRNA-1273 in 7% of participants). Characteristics of patients excluded from analyses are described in Supplementary Table S2. Healthy, age-matched, SARS-CoV-2-unexposed individuals who received a primary 2-dose mRNA-1273 vaccination schedule followed by 1 BNT162b booster vaccination served as a control cohort. ^#^Partially overlapping with other exclusion criteria.

**Fig. 2:**
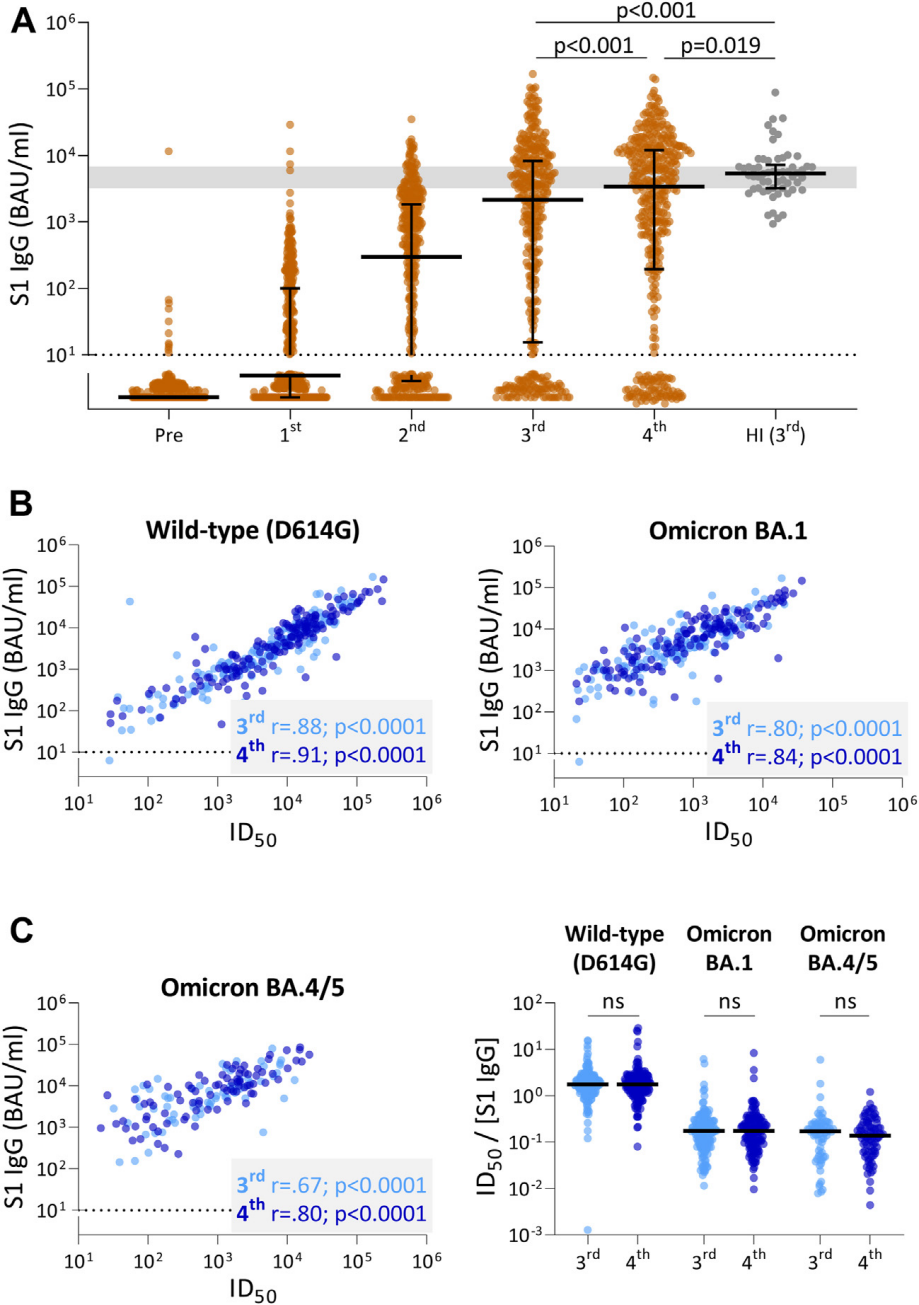
**Antibody concentration and virus neutralisation. A.** S1 IgG antibody concentration 4 weeks after each vaccination (second, third and fourth) in binding antibody units (BAU) per millilitre, where median and IQR are indicated by black lines. Grey bar indicates IQR of S1 IgG concentration (3219–6820 BAU/mL) in 3-dose vaccinated healthy individuals. **B.** Correlation between S1 IgG antibody concentration and pseudovirus neutralisation (ID_50_) of SARS-CoV-2 wild-type and Omicron variants after the third (light blue) and fourth (dark blue) vaccination. **C.** Antibody maturity, defined as SARS-CoV-2 wild-type and Omicron variant neutralising capacity per antibody after third (light blue) and fourth (dark blue) vaccination. Black lines indicate median values. Ns: Not significant, p > 0.05.

Between March 24, 2021 and May 4, 2021, 723 patients with haematological diseases were enrolled. The current analysis focussed on patients with a haematological malignancy, who remained SARS-CoV-2 naive throughout the period under study and received 4 mRNA vaccinations according to protocol. 414 participants fulfilled these inclusion criteria (Fig. 1; Table 1). 91 participants were excluded because of a previous SARS-CoV-2 infection (Supplementary Table S1). Only 1 study participant died as a result of COVID-19 (Fig. 1). Characteristics of study participants who did not meet the inclusion criteria are summarised in Supplementary Table S2. S1 IgG concentration increased significantly from 2144 BAU/mL (median; IQR 16–8248 BAU/mL) after the third vaccination to 3388 BAU/mL (median; IQR 195–11937 BAU/mL) after the fourth vaccination (p < 0.001). However, concentrations remained significantly lower than concentrations obtained by 3-dose vaccinated age-matched, healthy individuals (median 5375 BAU/mL, p = 0.019) (Table 1; Fig. 2A). The serum S1 IgG concentration correlated significantly with pseudovirus neutralising activity (ID_50_) for SARS-CoV-2 wild-type (r = 0.91; p < 0.0001) and Omicron variants (BA.1: r = 0.84; p < 0.0001; BA.4: r = 0.80; p < 0.0001) (Fig. 2B; Supplementary Table S3). The neutralisation capacity per antibody, expressed as the ratio of ID_50_ to S1 IgG concentration, that improved significantly after the third vaccination,^7^ did not further increase after the fourth vaccination, regardless of the SARS-CoV-2 variant (Fig. 2C).

**Table 1:** Baseline characteristics and S1 IgG concentrations 4 weeks after third and fourth COVID-19 vaccination, stratified by disease and treatment at time of first COVID-19 vaccination.

	n	Age	Women	Seroconversion^a^		S1 IgG serum concentration (BAU/mL)				
				3rd	4th	3rd	4th	4th vs. 3rd	3rd vs. HI^b^	4th vs. HI^b^
		Median (IQR)	n (%)	n (%)	n (%)	Median (IQR)	Median (IQR)	p	p	p
**All patients**	414	62 (56–68)	152 (37)	311 (77)	337 (81)	2144 (16–8248)	3388 (195–11,937)	**<0.001**	*<0.001*	*0.019*
**Lymphoma**										
During anti-CD20 therapy	27	62 (57–67)	12 (44)	6 (22)	11 (41)	1 (0–10)	2 (0–192)	**0.049**	*<0.001*	*<0.001*
Anti-CD20 therapy <12 mo	25	66 (56–72)	9 (36)	17 (71)	21 (84)	238 (4–2106)	1411 (479–3358)	0.145	*<0.001*	*<0.001*
BEAM-autologous HCT <12 mo	18	64 (58–66)	4 (22)	7 (41)	14 (78)	5 (1–13,565)	567 (13–13,350)	0.287	*0.007*	0.115
**CD19 CAR T cell therapy**	34	64 (57–69)	10 (29)	10 (30)	11 (32)	0 (0–60)	0 (0–380)	0.728	*<0.001*	*<0.001*
**CLL**										
Watch and wait	28	66 (59–70)	12 (43)	22 (85)	25 (89)	3796 (197–9315)	8233 (721–14,121)	**0.012**	0.202	0.652
Ibrutinib	23	67 (61–71)	9 (39)	13 (57)	14 (61)	44 (1–3352)	146 (1–958)	0.855	*<0.001*	*<0.001*
**Multiple myeloma**										
Induction therapy	15	65 (59–70)	7 (47)	14 (93)	14 (93)	5657 (111–12,341)	6307 (1411–11,485)	0.140	0.405	0.672
Daratumumab	37	66 (58–70)	15 (41)	35 (97)	36 (97)	1933 (962–3867)	2182 (1005–4176)	0.271	*<0.001*	*<0.001*
IMiD	32	61 (55–65)	14 (44)	27 (84)	28 (88)	3245 (738–7249)	4090 (590–11336)	0.079	*0.038*	0.262
HDM-autologous HCT <9 mo	31	62 (59–66)	9 (29)	30 (97)	30 (97)	8665 (5140–17,404)	13,737 (6915–32,616)	0.078	**0.007**	**<0.001**
**AML and high-risk MDS**										
Hypomethylating agents	6	73 (71–73)	3 (50)	6 (100)	5 (83)	1704 (212–3160)	3126 (321–9464)	**0.046**	*0.014*	0.289
High-dose chemotherapy	11	60 (49–64)	4 (36)	11 (100)	11 (100)	10,954 (4454–20,478)	25,912 (9976–48,083)	**0.004**	**0.029**	**0.003**
**MPN**										
Ruxolitinib	22	61 (50–67)	9 (41)	22 (100)	22 (100)	2304 (1005–4206)	3343 (1015–5170)	0.570	*<0.001*	*0.013*
**CML**										
Tyrosine kinase inhibitor	28	60 (52–66)	8 (29)	27 (100)	28 (100)	7572 (3992–11,120)	9472 (5195–14,301)	**0.002**	0.070	**0.003**
**Allogeneic HCT**										
<6 months	32	60 (54–67)	13 (41)	25 (81)	27 (84)	3769 (42–33,943)	9040 (910–23,887)	0.281	0.438	0.332
Chronic GvHD	37	59 (52–66)	10 (27)	33 (92)	34 (92)	4830 (497–21,143)	6784 (1535–14,216)	0.826	0.619	0.436
**Intercurrent cell therapy**^c^										
HDM-autologous HCT^d^	9	60 (59–66)	4 (44)	9 (100)	8 (89)	6108 (249–12,341)	6344 (1442–11,485)	0.214	0.633	0.869
Allogeneic HCT	5	62 (60–62)	1 (20)	4 (100)	4 (80)	56 (38–79)	115 (22–5782)	0.715	*<0.001*	0.140
CD19 CAR T cell therapy	3	65 (27–66)	3 (100)	2 (67)	2 (67)	514 (0–731)	104 (0–183)	0.109	*0.004*	*0.004*
**Healthy individuals (HI)**	58	62 (59–65)	34 (59)	58 (100)	N/A	5375 (3219–6820)	N/A	N/A	N/A	N/A

p values: bold indicates significantly higher and italic significantly lower compared to the comparative factor (p ≤ 0.05).

**Fig. 3:**
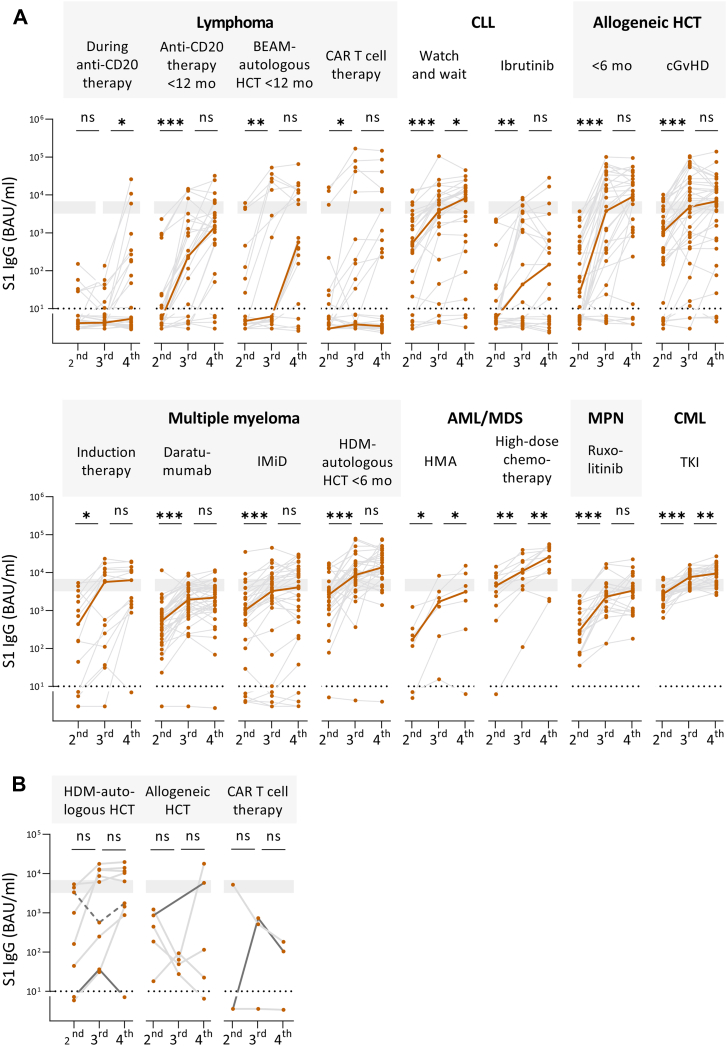
**Heterogeneity between cohorts. A.** S1 IgG concentrations 4 weeks after each vaccination (second, third and fourth) in binding antibody units (BAU) per millilitre, for each patient cohort. Orange lines indicate median values. Grey bar indicates IQR (3225–8939 BAU/mL) in 3-dose vaccinated, age-matched, healthy individuals (Fig. 2A). Dotted line indicates threshold for seroconversion (10 BAU/mL). Significances are shown for differences in antibody concentration between second and third vaccination response, and between third and fourth vaccination response. For statistical significance of difference between antibody concentrations after a fourth dose in patients and third dose in healthy individuals, see Table 1. Mo: months; BEAM: Carmustine, etoposide, cytarabine, melphalan; HCT: Haematopoietic cell transplantation; CAR: Chimeric antigen receptor; CLL: Chronic lymphocytic leukaemia; cGvHD: Chronic graft-versus-host disease; IMiD: Immunomodulatory imide drug; HDM: High-dose melphalan; AML: Acute myeloid leukaemia; MDS: Myelodysplastic syndrome; HMA: Hypomethylating agents; MPN: Myeloproliferative neoplasm; CML: Chronic myeloid leukaemia; TKI: Tyrosine kinase inhibitor; ns: p > 0.05; ∗: p ≤ 0.05; ∗∗: p ≤ 0.005; ∗∗∗: p ≤ 0.001. **B.** S1 IgG concentrations 4 weeks after each vaccination in patients who received cell therapy between the second and third vaccination (grey line), between third and fourth vaccination (black line), or who received a tandem transplantation (one after the second vaccination and one after the third vaccination; dashed line). HDM-autologous HCT: subgroup of multiple myeloma patients who received induction therapy at the time of the first COVID-19 vaccination. Grey bar indicates IQR of S1 IgG concentration (3219–6820 BAU/mL) in 3-dose vaccinated healthy individuals (Fig. 2A). Dotted line indicates threshold for seroconversion (10 BAU/mL). Ns: not significant, p > 0.05.

S1: Spike protein subunit 1; BAU: Binding antibody units; mo: months; BEAM: Carmustine, etoposide, cytarabine, melphalan; HCT: Haematopoietic cell transplantation; CAR: Chimeric antigen receptor; CLL: Chronic lymphocytic leukaemia; IMiD: Immunomodulatory imide drug; HDM: High-dose melphalan; AML: Acute myeloid leukaemia; MDS: Myelodysplastic syndrome; MPN: Myeloproliferative neoplasm; CML: Chronic myeloid leukaemia; cGvHD: Chronic graft-versus-host disease.

a Seroconversion: defined as S1 IgG >10 BAU/mL.

b HI: Healthy individuals.

c Intercurrent cell therapy: patients who received cell therapy between second and fourth vaccination.

d HDM-autologous HCT: subgroup of multiple myeloma patients who received induction therapy at time of first COVID-19 vaccination (see Fig. 3B).

### Heterogeneity between cohorts

While S1 IgG concentrations increased in the majority of patients after the fourth (first booster) vaccination, antibody concentrations varied considerably between patient subcohorts. We therefore analysed antibody dynamics over time per cohort (Table 1, Fig. 3A). A few of the subcohorts obtained antibody concentrations after 4 vaccinations (standard 3-dose vaccination scheme for immunocompromised patients plus first booster) that were actually significantly higher compared to healthy individuals after 3 vaccinations (standard primary vaccination scheme and first booster): these were patients with multiple myeloma who had received their first vaccination <9 months after high dose melphalan followed by autologous haematopoietic progenitor cell transplantation (HCT), patients with acute myeloid leukaemia (AML) who received their first vaccination during or <12 months after high-dose remission-induction chemotherapy, and tyrosine kinase inhibitor treated patients with chronic myeloid leukaemia (Table 1, Fig. 3A). In other subcohorts, S1 IgG concentrations were not significantly different than those obtained by healthy individuals after a first booster vaccination: patients with non-Hodgkin lymphoma (NHL) who received BEAM (carmustine, etoposide, cytarabine, melphalan) chemotherapy followed by autologous HCT less than 12 months before receiving the first vaccination, untreated patients with chronic lymphocytic leukaemia (CLL), patients with multiple myeloma who received remission-induction chemotherapy or immune modulating imide drugs (IMiDs) at the time of first vaccination, patients with AML using hypomethylating agents, and patients who received an allogeneic HCT less than 6 months before the first vaccination or patients with chronic graft versus host disease (GvHD) (Table 1, Fig. 3A). In 6 subcohorts, S1 IgG responses remained significantly lower than in healthy individuals: these were patients with B NHL who received the first vaccination during or shortly after completing CD20 antibody therapy, patients with CLL using ibrutinib with or without venetoclax, patients with multiple myeloma using daratumumab, patients who received CD19-directed chimeric antigen receptor (CAR) T cell therapy, and patients with myeloproliferative neoplasms using ruxolitinib (Table 1, Fig. 3A). Across cohorts, 63% (47/75) of the patients who did not seroconvert had no detectable circulating B cells (<1 cell/μL), while only 3% (2/75) of non-seroconverting patients had normal circulating B cell numbers (100–500 cells/μL) at the time of the fourth vaccination. B cell depletion was related to (a history of) the use of CD20 antibody therapy, CD19 CAR T cell therapy, low dose cyclophosphamide or delayed B cell recovery after allogeneic HCT. Despite 4 vaccinations, 39% (9/23) of CLL patients receiving ibrutinib did not seroconvert. None of these non-seroconverting CLL patients had undetectable circulating B cells, while the concomitant use of venetoclax was more frequent compared to CLL patients who did seroconvert (44% vs. 7%).

### Intercurrent cell therapy

A number of patients had received intercurrent cell therapy in the months between the second and the fourth vaccination. S1 IgG concentrations over time of these patients are depicted in Fig. 3B. In patients with multiple myeloma who received high dose melphalan and autologous HCT between the second and fourth vaccination (n = 9) build-up antibody concentrations were preserved. Median S1 IgG concentrations after the first booster (fourth vaccination) were even comparable to those obtained by healthy individuals after a first booster (third vaccination), despite intercurrent autologous HCT (Table 1, Fig. 3B). Of 5 patients who received allogeneic HCT after the second vaccination, 2 obtained normal S1 IgG concentrations after the first booster (fourth vaccination) (Table 1, Fig. 3B). Following intercurrent CAR T cell therapy (n = 3), antibody concentrations decreased despite booster vaccination (Table 1, Fig. 3B).

### Priming effect of vaccination during B cell depletion

**Fig. 4:**
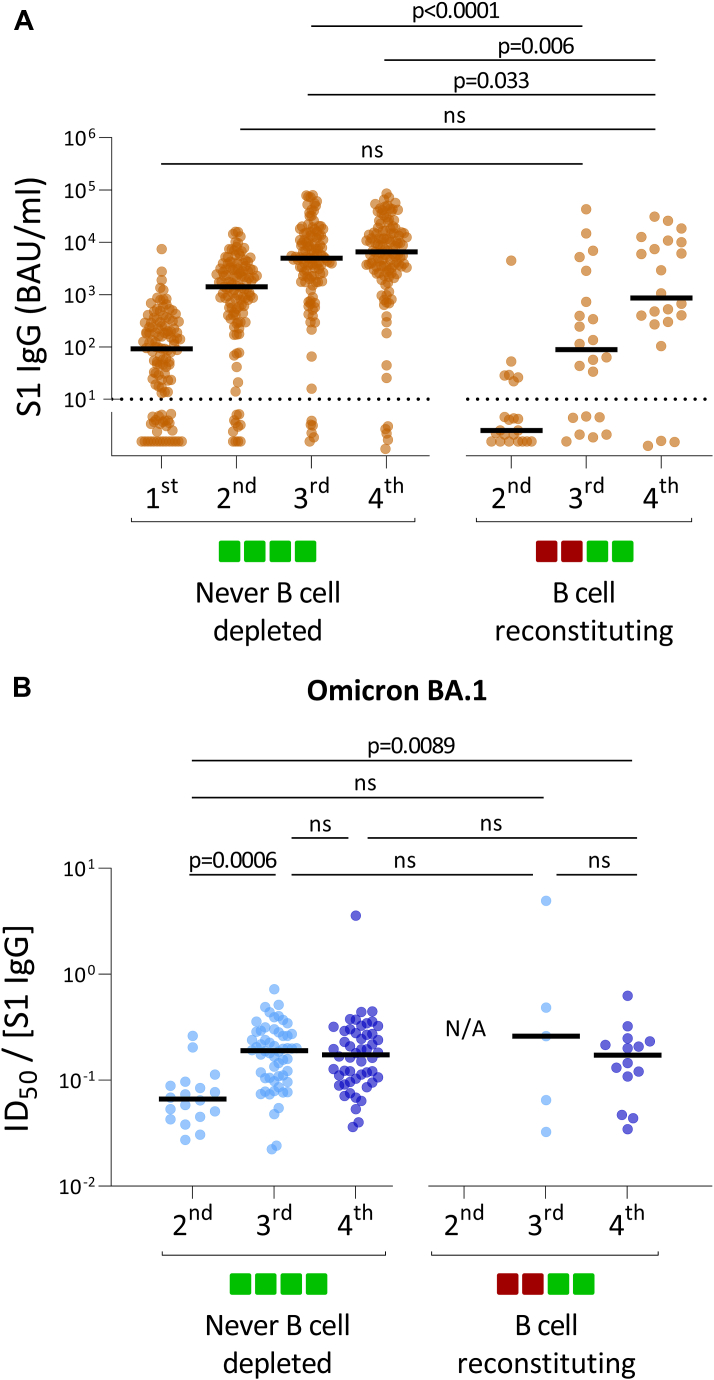
**Priming effect of vaccinations during B cell depletion. A.** S1 IgG concentration after each vaccination for patients with normal absolute B cell numbers (100–500 cells/μL) at the day of the first vaccination (‘never B cell depleted’; n = 119 (Supplementary Table S4)), and for patients who were B cell depleted (0 cells/μL) at the time of the primary 2-dose vaccination and had circulating B cells (≥1 cell/μL) from the third vaccination onwards (‘B cell reconstituting’; n = 22 (Supplementary Table S4)). **B.** Capacity per antibody to neutralise Omicron BA.1 (ratio of ID_50_ to the binding S1 IgG concentration) in never B cell depleted and B cell reconstituting patients. N/A: Not applicable.

The longitudinal set-up of our study allowed us to investigate the question whether vaccination during B cell depletion had a priming effect for antibody responses after subsequent B cell recovery. Twenty-two participants received the first and second vaccination when circulating B cell numbers were below the level of detection, but had measurable (not necessarily normal) B cell numbers at the time of the third vaccination (median B cell number (IQR): 35 (6–72) cells/μL at third vaccination; 72 (8-161) cells/μL at fourth vaccination). These included patients with B NHL who had completed anti-CD20 therapy (n = 13; 59%), allogeneic HCT recipients (n = 3; 14%), CD19-directed CAR T cell recipients in whom B cells had reappeared (n = 4; 18%; in n = 2 only temporarily), 1 patient with CLL on ibrutinib (5%), and 1 AML patient who received high-dose remission-induction chemotherapy at the time of the first vaccination (5%; Supplementary Table S4). These patients are referred to as ‘B cell-reconstituting patients’. We compared S1 IgG concentrations between these patients and patients with normal B cell numbers from the time of the first vaccination onwards (‘never B cell depleted patients’; n = 119; Supplementary Table S4). Of note, T cell numbers were lower in the ‘B cell reconstituting’ subgroup compared to the ‘never B cell depleted’ group, but none of the ‘B cell reconstituting’ patients had undetectable T cell numbers at any of the time points measured (Supplementary Table S5). In the B cell reconstituting patients, S1 IgG concentrations were very low to undetectable after the first and second vaccination, as expected (Fig. 4A). After the fourth vaccination, B cell reconstituting patients had obtained antibody concentrations that were significantly lower than obtained by never B cell depleted patients after 4 vaccinations. Rather, antibody concentrations were comparable to concentrations obtained by never B cell depleted patients after the second vaccination (Fig. 4A). Despite significantly lower antibody concentrations after 4 vaccinations in B cell reconstituting patients (Fig. 4A), the Omicron virus neutralising capacity per antibody reached after 4 vaccinations was similar as that observed in never B cell depleted patients after the fourth vaccination (Fig. 4B).

## Discussion

In this study we demonstrate that a fourth mRNA COVID-19 vaccination significantly improved neutralising antibody concentrations in a diverse cohort of immunocompromised patients with haematological malignancies. However, concentrations remained significantly lower than those obtained by 3-dose vaccinated healthy individuals and 19% of patients did not seroconvert at all, despite 4 vaccinations. The majority of non-seroconverters had undetectable circulating B cell numbers. This is in line with earlier reports on factors that impair antibody responses, including the use of B cell depleting therapy, delayed B cell reconstitution after allogeneic HCT, single or combined use of ibrutinib and venetoclax, daratumumab, ruxolitinib, and the use of more than 2 immunosuppressants.^6^^,^^11^^,^^12^^,^^22^^,^^23^ Low-dose cyclophosphamide in patients with multiple myeloma receiving IMiDs was also associated with lower antibody concentrations.

It has been demonstrated that in the absence of a humoral vaccination response, for example in patients with a lymphoid malignancy, vaccination can induce potent T cell immunity.^24^^,^^25^ This raised the question whether having such a primed cellular compartment would benefit patients who were B cell-depleted during the primary 2-dose vaccination schedule, but who received subsequent vaccinations during or after B cell reconstitution. Our data suggest that having a primed cellular compartment, as a result of vaccination at the time of B cell depletion, did not significantly enhance quantitative antibody responses when B cells reappeared. Nevertheless, having a primed cellular compartment seemed to support antibody maturation in response to subsequent vaccinations, a finding that needs to be confirmed in other studies. The state of B cell and antibody maturation is relevant as it is likely to contribute to the breadth of antibody reactivity following subsequent antigen encounters (virus or vaccination) and to the building of a long-lived, antibody secreting plasma cell population that confers lasting immunity.^26^ Whether having a lower amount of antibodies with better quality is associated with sufficient protection against COVID-19 morbidity and mortality needs to be confirmed in vaccine effectiveness studies. If confirmed, it has implications regarding the number of re-vaccinations B cell depleted patients may need. Together, these data make clear that it is important to vaccinate immunocompromised individuals, even when B cells are absent. In addition to the potential protection of cellular immunity against severe COVID-19,^27^ primed cellular immunity may enhance humoral immune responses when B cell numbers are restored. The number of booster vaccinations that these patients need to obtain full immunity remains to be determined.

Another remaining question is whether vaccine-induced SARS-CoV-2 immunity persists in patients who are diagnosed with a haematological malignancy and/or receive new treatments after vaccination. The long follow-up of our cohort allowed us to partially address this question. We measured antibody concentrations in study participants who received autologous or allogeneic HCT after the second vaccination. Most of these patients had obtained normal S1 IgG concentrations when compared to 2-dose vaccinated healthy individuals.^6^^,^^7^ In patients with multiple myeloma, high dose melphalan and autologous HCT did not negatively affect immunity or hamper further improvement of antibody concentrations following subsequent vaccinations. In allogeneic HCT recipients, S1 IgG antibody responses were more heterogeneous, with 2 out of 5 patients reaching antibody concentrations comparable to healthy individuals after a first booster.

Our study has a few limitations. The results of antibody concentration measurements were shared with study participants after each vaccination, and some participants deferred a third or fourth vaccination because they felt they had reached sufficiently high antibody concentrations after the primary series. On the other end of the spectrum, there were some non-seroconverters who felt demotivated to continue. Sickle cell patients included in our cohort did not receive a fourth vaccination during the period of follow-up and will be reported separately. Moreover, some subcohorts were of relatively small size and no correction for multiple testing was performed. Finally, the clinical relevance of differences in antibody concentrations cannot be determined in the absence of a correlate of protection.

In conclusion, a fourth mRNA COVID-19 vaccination further improved humoral responses in immunocompromised patients with haematological malignancies. In absence of B cells, mRNA vaccination primed the immune system to enhance antibody maturity after recovery of the B cell pool. Our data argue for continued vaccination, even in patients that are immunocompromised. Vaccine effectiveness studies that focus on patients with a specific type of malignancy and stage of therapy (e.g. during, early after and late after therapy) are required to determine the consequences of the observed suboptimal antibody responses on COVID-19-related morbidity and mortality for individual patients.

## Contributors

A.G., C.E.R., M.D.H. and I.S.N. initiated and designed the study; A.E.C.B., J.A.D., K.G., T.M., P.G.N.J.M., C.E.R., M.D.H. and I.S.N. recruited patients, and together with A.G. and S.V.-N. collected clinical data; B.I.L.-W., S.H. and Q.H. performed statistical analyses; Q.H., S.H., M.S.B., N.J.E.H., J.M., J.A.B., J.H.B., G.P.S., R.S.B., G.H., D.W., E.M.M.L., H.J.B., N.K., N.R., J.B., M.H.M.H., M.J.G. collected or supervised collection of experimental data; Q.H., S.H., B.I.L.-W., A.G., C.E.R., I.S.N. and M.D.H. have accessed and verified the data; Q.H., S.H., A.G., C.E.R., I.S.N. and M.D.H. wrote the first drafts of the manuscript; all authors contributed to and approved the final version; and I.S.N., C.E.R., A.G. and M.D.H. were responsible for the decision to submit the manuscript.

## Data sharing statement

For original data or de-identified individual participant data, please contact the corresponding author Prof. Dr. M.D. Hazenberg by sending an e-mail to m.d.hazenberg@amsterdamumc.nl. The study protocol is included as a data supplement available with the online version of this article.

## Declaration of interests

T.M. received research grants from Celgene/BMS, Genentech, and Siemens, and received consulting fees from Kite/Gilead, Janssen, Lilly (all payments made to institution). All other authors declare no competing financial interests.
