# Supplementary material for: Fourth mRNA COVID-19 vaccination in immunocompromised patients with haematological malignancies (COBRA KAI): a cohort study
Source: eClinicalMedicine. 2023 Jun 15;61:102040. doi: 10.1016/j.eclinm.2023.102040 (PMC10270678; doi:10.1016/j.eclinm.2023.102040)
Supplement: Supplementary Tables S1–S5 [file mmc1.docx]

**Supplementary Table 1**

| **Detection of infection** | **n (%)** |
| --- | --- |
| Baseline, i.e. prior to 1^st^ vaccination | 34 (37) |
| After baseline until 2^nd^ response measurement | 9 (10) |
| After 2^nd^ until 3^rd^ response measurement | 21 (23) |
| After 3^rd^ until 4^th^ response measurement | 27 (30) |
| *Total* | *91 (100)* |

**Supplementary Table 1: SARS-CoV-2 (breakthrough) infections.** Timing of infection of the 91 study participants who were SARS-CoV-2 infected prior to or after inclusion in the study.

**Supplementary Table 2**

|  |  |  |  | **Indication for exclusion from analyses** | | | |
| --- | --- | --- | --- | --- | --- | --- | --- |
|  |  |  |  |  |  |  |  |
|  |  | **Age** | **Women** | **Death** | **Discon-tinuation** | **No fourth vaccination or response measurement** | **N IgG >14.3 BAU/mL^1^** |
|  | n | Median (IQR) | n (%) | n (%) | n (%) | n (%) | n (%) |
|  |  |  |  |  |  |  |  |
| **All patients** | 309 | 58 (47-65) | 134 (43) | 42 (14) | 46 (15) | 170 (55) | 91 (29) |
|  |  |  |  |  |  |  |  |
| **Lymphoma** |  |  |  |  |  |  |  |
| During anti-CD20 therapy | 18 | 64 (58-66) | 7 (39) | 3 (17) | 3 (17) | 9 (50) | 5 (28) |
| Anti-CD20 therapy <12 mo | 15 | 65 (51-68) | 8 (53) | 3 (20) | 4 (27) | 6 (40) | 4 (27) |
| BEAM-autologous HCT <12 mo | 11 | 50 (39-64) | 5 (45) | 3 (27) | 3 (27) | 5 (45) | 1 (9) |
|  |  |  |  |  |  |  |  |
| **CD19 CAR T cell therapy** | 19 | 59 (49-68) | 7 (37) | 4 (21) | 6 (32) | 6 (32) | 5 (26) |
|  |  |  |  |  |  |  |  |
| **CLL** |  |  |  |  |  |  |  |
| Watch and wait | 28 | 67 (61-71) | 15 (54) | 2 (7) | 4 (14) | 19 (68) | 8 (29) |
| Ibrutinib | 14 | 63 (58-66) | 4 (29) | 3 (21) | 1 (7) | 6 (43) | 4 (29) |
|  |  |  |  |  |  |  |  |
| **Multiple myeloma** |  |  |  |  |  |  |  |
| Induction therapy | 13 | 58 (56-66) | 5 (38) | 2 (15) | 3 (23) | 6 (46) | 5 (38) |
| Daratumumab | 15 | 60 (54-64) | 4 (27) | 3 (20) | 2 (13) | 8 (53) | 4 (27) |
| IMiD | 23 | 60 (56-71) | 7 (30) | 2 (9) | 2 (9) | 12 (52) | 9 (39) |
| HDM-autologous HCT <9 mo | 20 | 62 (60-65) | 8 (40) | 3 (15) | 3 (15) | 7 (35) | 8 (40) |
|  |  |  |  |  |  |  |  |
| **AML and high-risk MDS** |  |  |  |  |  |  |  |
| Hypomethylating agents | 12 | 71 (60-73) | 1 (8) | 6 (50) | 1 (8) | 5 (42) | 0 (0) |
| Consolidation chemotherapy | 9 | 40 (25-62) | 6 (67) | 2 (22) | 1 (11) | 5 (56) | 2 (22) |
|  |  |  |  |  |  |  |  |
| **MPN** |  |  |  |  |  |  |  |
| Ruxolitinib | 15 | 56 (51-63) | 7 (47) | 0 (0) | 5 (33) | 7 (47) | 8 (53) |
|  |  |  |  |  |  |  |  |
| **CML** |  |  |  |  |  |  |  |
| Tyrosine kinase inhibitor | 24 | 51 (43-56) | 18 (75) | 0 (0) | 3 (13) | 17 (71) | 7 (29) |
|  |  |  |  |  |  |  |  |
| **Allogeneic HCT** |  |  |  |  |  |  |  |
| <6 months | 22 | 56 (43-59) | 8 (36) | 5 (23) | 1 (5) | 12 (55) | 4 (18) |
| Chronic GvHD | 20 | 56 (46-60) | 10 (50) | 1 (5) | 2 (10) | 11 (55) | 8 (40) |
|  |  |  |  |  |  |  |  |
| **Sickle cell disease** |  |  |  |  |  |  |  |
| Hydroxyurea | 31 | 35 (28-47) | 14 (45) | 0 (0) | 2 (6) | 29 (94) | 9 (29) |

**Supplementary Table 2:** **Characteristics of study participants excluded from analyses.** ^1^Partially overlapping with other exclusion criteria. N: nucleocapsid; BAU: binding antibody units; mo: months; BEAM: carmustine, etoposide, cytarabine, melphalan; HCT: hematopoietic cell transplantation; CAR: chimeric antigen receptor; CLL: chronic lymphocytic leukaemia; IMiD: immunomodulatory imide drug; HDM: high-dose melphalan; AML: acute myeloid leukaemia; MDS: myelodysplastic syndrome; MPN: myeloproliferative neoplasm; CML: chronic myeloid leukaemia; GvHD: graft-versus-host disease.

**Supplementary Table 3**

|  |  | **Age** | **Women** | **Seroconversion^1^** | | **S1 IgG (BAU/mL)** | |
| --- | --- | --- | --- | --- | --- | --- | --- |
|  |  |  |  | **Third** | **Fourth** | **Third** | **Fourth** |
|  | n | Median (IQR) | n (%) | n (%) | n (%) | Median (IQR) | Median (IQR) |
|  |  |  |  |  |  |  |  |
| **All patients** | 164 | 63 (56-69) | 59 (36) | 150 (91) | 163 (99) | 2999 (348-8807) | 4559 (1007-12516) |
|  |  |  |  |  |  |  |  |
| **Lymphoma** |  |  |  |  |  |  |  |
| During anti-CD20 therapy | 10 | 63 (59-67) | 3 (30) | 5 (50) | 10 (100) | 12 (1-44) | 309 (175-5943) |
| Anti-CD20 therapy <12 months | 18 | 66 (53-71) | 8 (44) | 14 (78) | 18 (100) | 588 (63-3227) | 1766 (672-6424) |
| BEAM-autologous HCT <12 months | 11 | 64 (55-70) | 3 (27) | 7 (64) | 11 (100) | 2852 (2-27161) | 7424 (401-17918) |
|  |  |  |  |  |  |  |  |
| **CD19 CAR T cell therapy** | 10 | 64 (53-70) | 1 (10) | 10 (100) | 10 (100) | 6328 (155-53952) | 8243 (641-41117) |
|  |  |  |  |  |  |  |  |
| **CLL** |  |  |  |  |  |  |  |
| Watch and wait | 9 | 68 (60-71) | 4 (44) | 9 (100) | 9 (100) | 2784 (1460-7581) | 7833 (2694-11967) |
| Ibrutinib | 10 | 69 (61-73) | 3 (30) | 10 (100) | 10 (100) | 2761 (1207-5420) | 798 (398-5996) |
|  |  |  |  |  |  |  |  |
| **Multiple myeloma** |  |  |  |  |  |  |  |
| Induction therapy | 6 | 67 (65-70) | 3 (50) | 5 (83) | 6 (100) | 3034 (111-10158) | 4227 (1411-10818) |
| Daratumumab | 11 | 60 (54-66) | 5 (45) | 11 (100) | 11 (100) | 1729 (351-4988) | 2182 (596-6608) |
| IMiD | 10 | 62 (56-62) | 4 (40) | 10 (100) | 10 (100) | 2369 (1504-4039) | 3203 (1020-7703) |
| HDM-autologous HCT <9 months | 8 | 65 (59-68) | 3 (38) | 8 (100) | 8 (100) | 8244 (4292-12223) | 10202 (6017-32810) |
|  |  |  |  |  |  |  |  |
| **AML and high-risk MDS** |  |  |  |  |  |  |  |
| Hypomethylating agents | 6 | 73 (71-73) | 3 (50) | 6 (100) | 5 (83) | 1704 (212-3160) | 3126 (321-9464) |
| Consolidation chemotherapy | 10 | 60 (49-62) | 3 (30) | 10 (100) | 10 (100) | 11514 (6868-20478) | 32264 (11937-48083) |
|  |  |  |  |  |  |  |  |
| **MPN** |  |  |  |  |  |  |  |
| Ruxolitinib | 10 | 57 (50-67) | 3 (30) | 10 (100) | 10 (100) | 1487 (725-2894) | 3488 (893-3925) |
|  |  |  |  |  |  |  |  |
| **CML** |  |  |  |  |  |  |  |
| Tyrosine kinase inhibitor | 10 | 60 (52-65) | 3 (30) | 10 (100) | 10 (100) | 6191 (4161-8637) | 8981 (5406-9842) |
|  |  |  |  |  |  |  |  |
| **Allogeneic HCT** |  |  |  |  |  |  |  |
| <6 months | 13 | 61 (57-69) | 6 (46) | 13 (100) | 13 (100) | 12651 (395-33943) | 18207 (10346-26416) |
| Chronic GvHD | 11 | 57 (52-68) | 3 (27) | 11 (100) | 11 (100) | 3392 (760-12231) | 5776 (1535-11844) |
|  |  |  |  |  |  |  |  |
| **Intercurrent cell therapy** |  |  |  |  |  |  |  |
| Allogeneic HCT | 0 | N/A | N/A | N/A | N/A | N/A | N/A |
| CD19 CAR T cell therapy | 1 | 65 (65-65) | 1 (100) | 1 (100) | 1 (100) | 731 (731-731) | 104 (104-104) |

**Supplementary Table 3:** **Characteristics and S1 IgG serum concentrations of patients selected for antibody neutralising activity analysis.** ^1^Seroconversion: S1 IgG > 10 BAU/mL. S1: spike protein subunit 1; BAU: binding antibody units; BEAM: carmustine, etoposide, cytarabine, melphalan; HCT: hematopoietic cell transplantation; CAR: chimeric antigen receptor; CLL: chronic lymphocytic leukaemia; IMiD: immunomodulatory imide drug; HDM: high-dose melphalan; AML: acute myeloid leukaemia; MDS: myelodysplastic syndrome; MPN: myeloproliferative neoplasm; CML: chronic myeloid leukaemia; GvHD: graft-versus-host disease.

**Supplementary Table 4**

|  | **Number of patients** | | **Age (years)** | | **Women** | | **B cells (cells/µl)*** | |
| --- | --- | --- | --- | --- | --- | --- | --- | --- |
|  | n | | Median (IQR) | | n (%) | | Median (IQR) | |
|  | ■■■■ | ■■■■ | ■■■■ | ■■■■ | ■■■■ | ■■■■ | ■■■■ | ■■■■ |
| B cell depletion | Never | Reconstituting | Never | Reconstituting | Never | Reconstituting | Never | Reconstituting |
|  |  |  |  |  |  |  |  |  |
| **All patients** | 119 | 22 | 61 (55-66) | 65 (60-69) | 37 (31) | 11 (50) | 181 (102-280) | 72 (8-161) |
|  |  |  |  |  |  |  |  |  |
| **Lymphoma** |  |  |  |  |  |  |  |  |
| During aCD20 therapy | 1 | 4 | 45 (45-45) | 61 (53-63) | 1 (100) | 1 (25) | 0 (0-0) | 105 (60-205) |
| Anti-CD20 therapy <12 mo | 1 | 6 | 72 (72-72) | 69 (64-71) | 0 (0) | 4 (67) | 870 (870-870) | 73 (70-165) |
| BEAM-autologous HCT <12 mo | 3 | 2 | 55 (52-59) | 63 (57-68) | 0 (0) | 2 (100) | 387 (308-394) | 90 (55-125) |
|  |  |  |  |  |  |  |  |  |
| **CD19 CAR T cell therapy** | 2 | 4 | 69 (67-70) | 71 (66-72) | 0 (0) | 2 (50) | 391 (296-487) | 1 (1-6) |
|  |  |  |  |  |  |  |  |  |
| **CLL** |  |  |  |  |  |  |  |  |
| Watch and wait | 1 | 0 | 52 (52-52) | N/A | 1 (100) | N/A | 265 (265-265) | N/A |
| Ibrutinib | 8 | 1 | 70 (61-72) | 61 (61-61) | 3 (38) | 0 (0) | 165 (133-201) | 0 (0-0) |
|  |  |  |  |  |  |  |  |  |
| **Multiple myeloma** |  |  |  |  |  |  |  |  |
| Induction therapy | 0 | 0 | N/A | N/A | N/A | N/A | N/A | N/A |
| Daratumumab | 9 | 0 | 65 (60-72) | N/A | 4 (44) | N/A | 100 (46-238) | N/A |
| IMiD | 11 | 0 | 59 (54-62) | N/A | 3 (27) | N/A | 160 (137-240) | N/A |
| HDM-autologous HCT <9 mo | 14 | 0 | 63 (59-66) | N/A | 5 (36) | N/A | 108 (70-287) | N/A |
|  |  |  |  |  |  |  |  |  |
| **AML and high-risk MDS** |  |  |  |  |  |  |  |  |
| Hypomethylating agents | 0 | 0 | N/A | N/A | N/A | N/A | N/A | N/A |
| Consolidation therapy | 7 | 1 | 60 (56-62) | 65 (65-65) | 1 (14) | 1 (100) | 380 (332-386) | 69 (69-69) |
|  |  |  |  |  |  |  |  |  |
| **MPN** |  |  |  |  |  |  |  |  |
| Ruxolitinib | 15 | 0 | 61 (53-64) | N/A | 5 (33) | N/A | 200 (174-243) | N/A |
|  |  |  |  |  |  |  |  |  |
| **CML** |  |  |  |  |  |  |  |  |
| Tyrosine kinase inhibitor | 21 | 0 | 59 (52-63) | N/A | 6 (29) | N/A | 152 (104-188) | N/A |
|  |  |  |  |  |  |  |  |  |
| **Allogeneic HCT** |  |  |  |  |  |  |  |  |
| <6 months | 6 | 3 | 63 (59-68) | 61 (59-65) | 2 (33) | 0 (0) | 309 (179-415) | 200 (158-310) |
| Chronic GvHD | 18 | 0 | 61 (56-68) | N/A | 5 (28) | N/A | 189 (93-365) | N/A |
|  |  |  |  |  |  |  |  |  |
| **Intercurrent cell therapy** |  |  |  |  |  |  |  |  |
| Allogeneic HCT | 1 | 0 | 62 (62-62) | N/A | 0 (0) | N/A | 0 (0-0) | N/A |
| CD19 CAR T cell therapy | 1 | 1 | 66 (66-66) | 65 (65-65) | 1 (100) | 1 (0) | 0 (0-0) | 0 (0-0) |

**Supplementary Table 4:** **Characteristics of ‘never B cell depleted’ and ‘B cell reconstituting’ patients.** *Number of circulating B cells at time of fourth vaccination (normal values 100-500 cells/µL). Mo: months; BEAM: carmustine, etoposide, cytarabine, melphalan; HCT: hematopoietic cell transplantation; CAR: chimeric antigen receptor; CLL: chronic lymphocytic leukaemia; IMiD: immunomodulatory imide drug; HDM: high-dose melphalan; AML: acute myeloid leukaemia; MDS: myelodysplastic syndrome; MPN: myeloproliferative neoplasm; CML: chronic myeloid leukaemia; GvHD: graft-versus-host disease.

**Supplementary Table 5**

| **B cell depletion** | **■■■■**  **Never** | **■■■■**  **Reconstituting** | **p value** |
| --- | --- | --- | --- |
| **CD3+ T cells (cells/μl) – median (IQR)** |  |  |  |
| at 1^st^ vaccination | 996 (677-1540) | 603 (311-848) | 0.0021 (**) |
| at 2^nd^ vaccination | 984 (679-1487) | 641 (177-1331) | 0.0315 (*) |
| at 3^rd^ vaccination | 869 (627-1376) | 725 (428-1216) | 0.0440 (*) |
| at 4^th^ vaccination | 796 (608-1320) | 672 (391-903) | 0.0322 (*) |
| **CD4+ T cells (cells/μl) – median (IQR)** |  |  |  |
| at 1^st^ vaccination | 432 (280-744) | 370 (121-466) | 0.0336 (*) |
| at 2^nd^ vaccination | 438 (268-748) | 474 (121-644) | 0.1976 (ns) |
| at 3^rd^ vaccination | 446 (298-686) | 394 (214-621) | 0.1329 (ns) |
| at 4^th^ vaccination | 430 (271-637) | 361 (207-561) | 0.1831 (ns) |
| **CD8+ T cells (cells/μl) – median (IQR)** |  |  |  |
| at 1^st^ vaccination | 392 (270-698) | 187 (84-517) | 0.0048 (**) |
| at 2^nd^ vaccination | 392 (251-658) | 202 (96-688) | 0.0435 (*) |
| at 3^rd^ vaccination | 359 (227-601) | 225 (105-530) | 0.0577 (ns) |
| at 4^th^ vaccination | 309 (220-640) | 211 (88-456) | 0.0254 (*) |

**Supplementary Table 5:** **T cell numbers of ‘never B cell depleted’ and ‘B cell reconstituting’ patients.** P value indicates significance of difference between never B cell depleted and B cell reconstituting patients (ns: p>0.05; *: p≤0.05; **: p≤0.005).
